# Percutaneous Left Main Coronary Intervention: A Review of Plaque Modification in Left Main Percutaneous Coronary Intervention

**DOI:** 10.3390/jcm7070180

**Published:** 2018-07-23

**Authors:** Chirag A. Shah, Steven E. Pfau

**Affiliations:** 1 Yale-New Haven Hospital, Yale School of Medicine, New Haven, CT 06510, USA; 2 West Haven Veterans Administration Hospital, West Haven, CT 06516, USA

**Keywords:** left main coronary artery, left main percutaneous coronary intervention, coronary artery bypass surgery, atherectomy, percutaneous coronary intervention

## Abstract

Left main coronary artery (LMCA) stenosis has long been recognized as a marker of increased morbidity and mortality. Current treatment algorithms for LMCA stenosis consider both percutaneous coronary intervention (PCI) with drug eluting stents (DES) and coronary bypass surgery, each with advantages based on individual patient characteristics. Since the LMCA is the largest artery in the coronary tree, plaque volume and calcification is greater than other coronary segments and often extends to the distal bifurcation segment. In LMCA bifurcation lesions, larger minimal stent area is strongly associated with better outcome in the DES era. Plaque modification strategies such as rotational, orbital, or laser atherectomy are effective mechanisms to reduce plaque volume and alter compliance, facilitating stent delivery and stent expansion. We present a case of a calcified, medina class 1,1,1 LMCA lesion where intravascular ultrasound (IVUS) and orbital atherectomy were employed for optimal results. In this context, we review the evidence of plaque modification devices and the rationale for their use in unprotected left main PCI.

## 1. Introduction

Stenosis of the left main coronary artery (LMCA) is associated with increased morbidity and mortality in patients with coronary artery disease, when compared to elsewhere in the coronary tree [1]. Initial experiences with percutaneous treatment, including Grüntzig’s third patient, were associated with excess complications and mortality; as a result, surgical revascularization became firmly established as the standard of care [2]. With experience, improved stent technology, and important randomized trials, outcomes of percutaneous coronary intervention (PCI) with second generation drug-eluting stents (DES) may be similar to surgery in many patients, and PCI is the preferred strategy in patients with increased surgical risk [3,4,5,6,7,8,9,10]. Due to the size of the left main, plaque formation in the LMCA has a more disseminated distribution, higher plaque volumes, and an increased presence of severe calcification [11,12] when compared to lesions elsewhere in the coronary tree. Angioplasty and stenting for left main bifurcation stenosis, the most frequent phenotype of left main disease, therefore has important technical considerations compared to interventions in other coronary lesions. Intravascular ultrasound (IVUS) has contributed significantly to the understanding of procedural success in left main PCI, including that larger stent area is a significant predictor of improved long-term outcome, even in the DES era [13]. Atherectomy, using rotational, orbital, or laser devices is recognized as an effective mechanism for modification of plaque that can facilitate stent delivery and expansion, particularly in calcified lesions [14,15,16,17]. Application of debulking/plaque modification strategies may be particularly relevant in unprotected left main (UPLM) PCI where plaque volume is high, significant calcification is frequent, and final stent area is critical [11,12]. These considerations are particularly relevant as overlapping and stent crushing strategies are emerging as superior to single stent strategies in true bifurcation lesions [5,18]. Here we review the evidence for plaque modification strategies and the rationale for their more routine use in left main PCI.

## 2. Left Main PCI

**Figure 1:**
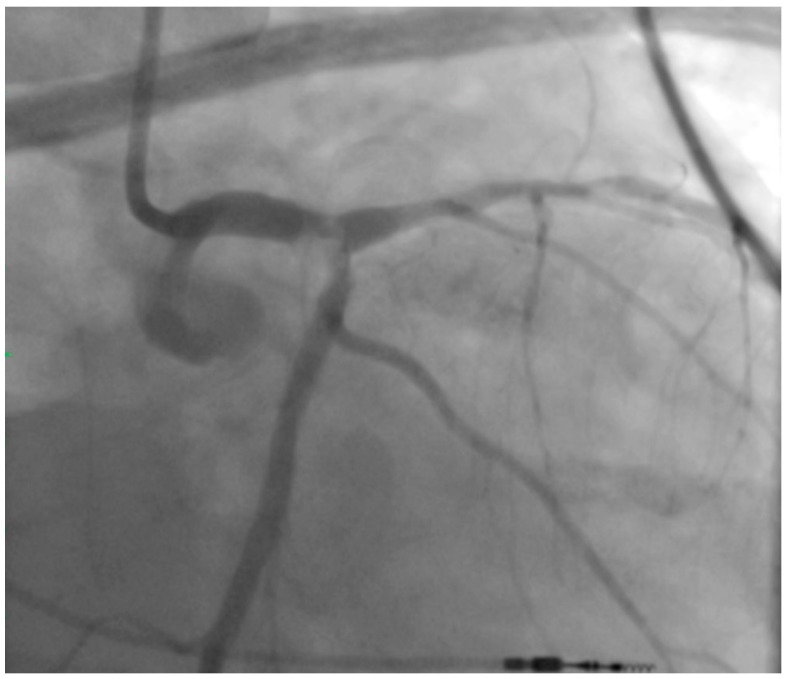
Pre-Intervention Coronary Angiogram. There is evidence of a calcific 80% distal left main coronary artery lesion that extends to the left anterior descending and left circumflex arteries (Medina 1.1.1).

A 79-year-old male presented for an elective coronary angiogram for Canadian Cardiovascular Society Class 3 angina and progressive dyspnea on exertion. Medical history was complex and included atrial fibrillation, insulin dependent diabetes, dual chamber pacemaker, prostate cancer, moderately differentiated adenocarcinoma of the ascending colon with a recent hemicolectomy, and heart failure with preserved ejection fraction. On coronary angiogram (Figure 1), he was found to have 80% distal LMCA stenosis extending to the left anterior descending (LAD) artery and Circumflex (CFX) artery (Medina 1.1.1), with moderate calcification. The right coronary was chronically occluded; the SYNTAX score was 42. A transthoracic echocardiogram revealed a preserved left ventricular ejection fraction of 55% and moderate aortic stenosis (peak velocity of 2.8 m/s, mean gradient of 18 mmHg, and calculated aortic valve area of 1.0 cm^2^). Cardiac surgery was consulted for coronary artery bypass surgery and aortic valve replacement, but he was deemed high risk for surgery due to a Society of Thoracic Surgery score of 11.6% and a heavily calcified ascending aorta. Following a Heart Team discussion, the decision was made to proceed with UPLM PCI.

Left main coronary artery disease is a complex disease process that is associated with increased morbidity and mortality due to the extent of myocardium that is at risk for ischemia [1,5,19,20]. For most patients with left main coronary artery disease, American College of Cardiology/American Heart Association practice guidelines recommend coronary artery bypass surgery (CABG) as the preferred mode of revascularization [21]. Contemporary randomized trials, however, suggest clinical equipoise for PCI and CABG in select patient populations [3,7,8,22,23] Five-year outcomes from the SYNTAX trial show that PCI for UPLM with a SYNTAX score <33 had similar rates of death, myocardial infarction, or stroke when compared to CABG [22], even though this trial utilized early generation DES. More recently, the EXCEL trial randomized 1905 subjects to PCI with a second-generation DES or CABG for UPLM stenosis with a low or intermediate SYNTAX score. In EXCEL, UPLM PCI with a SYNTAX score <33 was noninferior to CABG with respect to death, MI, or stroke at 3 years (15.4% for PCI versus 14.7% for CABG) [3]. Further, there was no difference in the Seattle Angina Questionnaire score at 1 year or 3 years [4]. The contemporaneous NOBLE trial randomized 1201 patients to either PCI (also with second generation DES) or CABG for LMCA stenosis with an intermediate SYNTAX score. In this study, CABG was superior to PCI with regards to MACCE at 5 years (28% for PCI and 18% for CABG, HR 1.51 with 95% CI 1.13–2.00 with a *p*-value of 0.0044) [23]. A recent meta-analysis, which examined 4499 subjects from 5 randomized studies (EXCEL, NOBLE, LE MANS, SYNTAX, and PRECOMBAT) suggested no difference between PCI and CABG for the treatment of UPLM disease for the composite endpoints of death, stroke, and MI (OR of 1.03, 95% CI 0.81–1.32, *p*-value of 0.81) [24]. While these trials enrolled patients with acceptable surgical risk, in patients with excess surgical risk, such as the case presented here, clearly UPLM PCI would be the preferred revascularization strategy.

## 3. Technical Considerations: The Role of Intravascular Ultrasound in UPLM PCI

Utilization of intracoronary imaging in PCI (particularly IVUS) has provided insights into coronary anatomy, plaque distribution and content, and mechanisms of PCI success and failure. This information has helped to select treatment strategies, and define optimal final stent diameter/area associated with improved PCI outcomes in the bare metal stent and DES eras [25,26,27]. In left main PCI, pre-procedural IVUS is critical for determining the severity of disease in both the proximal LAD artery and the proximal CFX artery [3,26,28]; the distribution of plaque in each branch will help to determine which will be the parent vessel (most commonly the LAD) and whether a single stent can be used. Angiography often underestimates calcification, and pre-intervention imaging can be an important tool to document whether calcification may be a factor in limiting complete stent expansion [28,29]. Additional information includes the distal reference vessel diameter and the length of stent that is required. Following stent deployment, IVUS is important to assure adequate stent expansion. In UPLM PCI, IVUS determined stent minimal lumen area (MLA) targets in the proximal LAD (6 mm^2^), proximal CFX (5 mm^2^), and distal LM (8 mm^2^) have been associated with the best short and intermediate term outcomes [13]. Published data regarding IVUS guidance in PCI for UPLM lesions is consistent in terms of improved mortality, rates of stent thrombosis, and event free survival when compared to LMCA revascularization without IVUS in the DES era [26,27,30]. In MAIN-COMPARE, IVUS guided UPLM PCI was associated with a 60% lower 3-year mortality rate when compared to angiographic guidance [27]. IVUS-guided PCI of the LMCA results in lower incidence of stent underexpansion, thus lower rates of in-stent restenosis (ISR) and target lesion revascularization (TLR) [30]. In this context, IVUS guidance has become standard practice for this lesion subset; approximately 80% of procedures in the EXCEL trial incorporated IVUS as an adjunctive tool [3].

**Figure 2:**
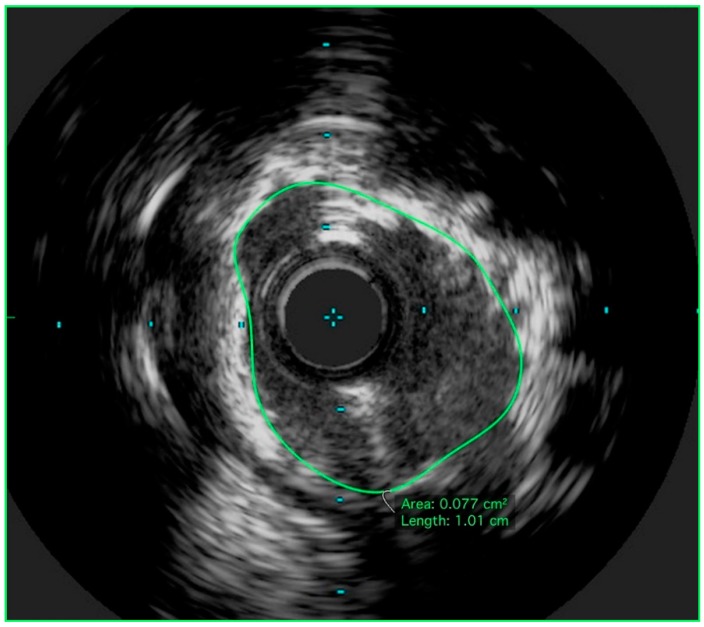
Post-orbital atherectomy IVUS of LMCA. IVUS of the LMCA with a 40 MHz imaging catheter performed after orbital atherectomy revealing 270 degrees of calcium and a minimal luminal area of 7.7 mm^2^. IVUS: Intravascular ultrasound; LMCA: Left main coronary artery.

The left coronary was engaged with an 8Fr extra-back up (EBU) 3.75 guide. By angiography, the LMCA was moderately calcified with extension of the calcium into the proximal LAD and CFX. Given the severity of the stenosis, obvious calcification, and right coronary artery CTO, a preintervention IVUS was deferred. The LMCA and LAD lesions were primarily wired with an orbital atherectomy guidewire. Orbital atherectomy was performed of the LMCA and proximal LAD using an orbital atherectomy catheter. One pass was made at 80,000 RPM (1.25 mm orbit), with 2 subsequent passes made at 120,000 RPM (3.0 mm orbit). Following atherectomy, IVUS was performed using a 40 MHz imaging catheter, revealing a 270-degree arc of calcium in the distal LMCA (Figure 2) with an MLA of 7.7 mm^2^. At this point, the CFX lesion was crossed using an 0.014 mm coronary guidewire. A 40 MHz IVUS was then used to interrogate the CFX, revealing 35 degrees of dense calcification. Given the relative limited extent of calcification, atherectomy was not performed in the CFX.

## 4. Achieving Adequate Left Main Minimal Luminal Area

While stent area by IVUS is associated with improved short and long term outcome in the bare metal stent era [31,32], that association has been lost with the routine use of DES. Only a single randomized study (IVUS XPL) has been able to show a benefit from IVUS guided stent optimization of DES, specifically in long (greater than 28 mm) and small (less than 2.5 mm diameter) artery segments [33]. Although there are no randomized data, routine use of IVUS has been associated with improved outcome in UPLM PCI, and adequate stent expansion is one mechanism by which routine IVUS accomplishes this difference. In registry data, stent underexpansion with current generation DES is an independent predictor of 2-year major adverse cardiac events (MACE) and TLR [13,29]. In contrast, acute stent malapposition in UPLM PCI was not related to ISR or MACE [13]. The group at Asan Medical Center in Seoul, Korea has provided the most systematic analysis of the LMCA IVUS information. For the purposes of their retrospective outcomes study, the LMCA is divided into 4 segments: (1) the ostial left circumflex artery (5 mm distal to the carina); (2) the ostial left anterior descending artery (5 mm distal to the carina); (3) the polygon of confluence (POC, the confluence zone of the LAD and LCx on longitudinal IVUS image reconstruction); and (4) the proximal left main segment (just above the POC) [13]. In UPLM PCI, a smaller IVUS-derived minimal stent area (MSA) in any of those segments predict the development of angiographic ISR at 9 months. In this study, the IVUS-minimal stent area with the lowest ISR and 2-year MACE were 5.0 mm^2^ for the LCx ostium, 6.3 mm^2^ for the LAD ostium, 7.2 mm^2^ for the POC, and 8.2 mm^2^ for the proximal LM above the POC. UPLM PCI can be approached via varying techniques, either requiring 1-stent or 2-stents. DKCRUSH V has shown that the 2-stent technique has lower rates of target lesion failure (TLF) at 1 year when compared to single stent technique [18]. While 2-stent techniques had a higher incidence of stent underexpansion when compared to the single stent technique [13], the predictive value of the IVUS areas was independent of the number of stents used. In this series of 400 cases, the most common site of stent underexpansion is the LCx ostia. Therefore, assuring adequate stent area through routine use of imaging is critical to obtaining the best procedural and clinical results in UPLM PCI [34,35].

## 5. Atherectomy in UPLM PCI

One of the most important predictors of stent underexpansion in any coronary lesion is the degree of coronary artery calcification [36,37,38]. Calcium acts to reduce compliance, thus limiting vessel expansion and limiting the effectiveness of balloon dilation, resulting in poor vessel preparation prior to stent deployment [37,38,39]. Accordingly, PCI in severely calcified lesions is associated with lower success rate, higher complication rates, and poor long-term outcomes when compared to non-calcified lesions [29,38,40]. In non-LMCA PCI, the use of atherectomy in calcific coronary disease prior to DES implantation is associated with higher procedural success rates, reduced incidence of ISR, and lower complications [17,41,42]. The mechanism of improved lesion compliance after atherectomy is not entirely clear, but it is unlikely that lesion modification is solely related to the volume of plaque removed or ablated.

Utilization of atherectomy in LMCA disease has been reported from several registries. In the ROTATE Registry, a registry of the utilization of rotational atherectomy, LMCA stenosis was the treatment target more commonly in older patients and those with diabetes, when compared to non-LMCA targets [43]. In the DELTA Registry of UPLM PCI, rotational atherectomy was used in 6.1% of lesions; the use of this technique was a predictor of the primary endpoint of death, MI and CVA (HR 1.73), as well as MACCE (HR 1.87) [44]. In neither of these observational studies were the reasons for use of rotational atherectomy identified, but likely use of this technique was a marker of higher lesion complexity and extensive coronary artery disease. In smaller studies, rotational atherectomy for calcified LMCA was associated with <20% TLR at 2 years [45,46]. Similarly, first reports of treatment of calcified LMCA stenosis using orbital atherectomy have demonstrated that it is technically feasible with a good short term outcome [14]. IVUS data of the LMCA cases has not been published from any of these atherectomy registries.

Interestingly, debulking of non-calcified LMCA disease has been reported in the bare metal stent era [47]. Directional atherectomy with stenting was associated with lower restenosis rates compared to stenting alone, even though MSA was not significantly larger. The authors hypothesize that residual plaque influenced restenosis, and atherectomy allowed lower residual plaque volumes. Similarly, residual plaque volume has been related to outcome after PCI with DES. Pre-procedural plaque volume as well as residual plaque volume after stent placement [48] are related to clinical outcome after DES, but not minimal stent area. Therefore, in addition to changing compliance in calcified lesions, there may be benefits to debulking beyond altering compliance and achieving the largest stent area.

Future therapies for plaque modification for calcified LMCA disease include coronary lithoplasty, a technique based on lithotripsy for renal calculi, where multiple emitters mounted on a balloon catheter create a diffusive, circumferential, pulsatile mechanical energy that disrupts calcified plaque [49,50]. Limited evidence for coronary lithoplasty is available, however optical coherence tomography (OCT) analysis has shown that lithoplasty improves lesion compliance in the presence of severe calcification by creating multiple fractures in single cross sections, which allows for complete stent expansion throughout the entire lesion [50]. The evidence for coronary lithoplasty is still limited and the current studies have not analyzed LMCA lesions. Large, randomized trials are still needed to establish the safety and efficacy of lithoplasty when compared to atherectomy devices, but conceptually this technology is attractive for LMCA disease.

**Figure 3:**
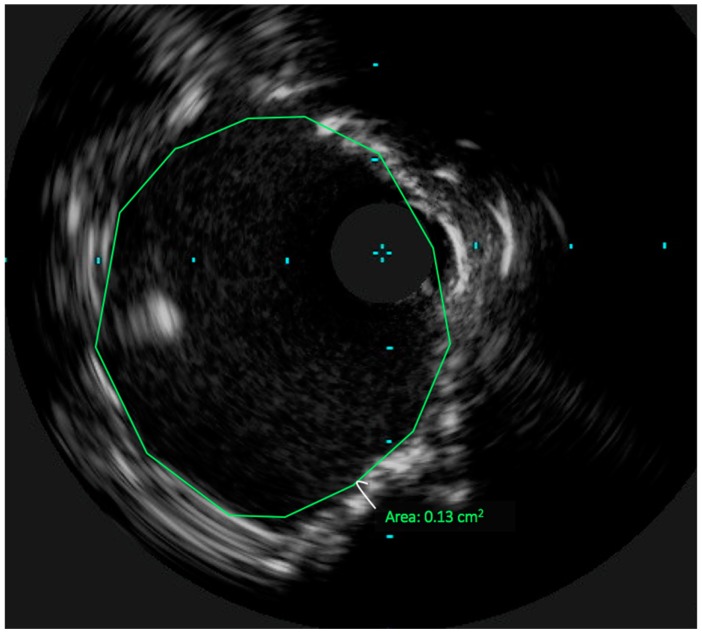
Post-PCI of the LMCA. IVUS of the LMCA after PCI with a 3.5 × 15 mm Resolute Onyx DES which dilated with a 4.0 mm noncompliant balloon. The minimal luminal area measures to 13 mm^2^. The CFX guidewire is just emerging into the LMCA lumen at 9 o’clock, which suggests that this image is in the distal LMCA. PCI: percutaneous coronary intervention. CFX: Left Circumflex Artery.

**Figure 4:**
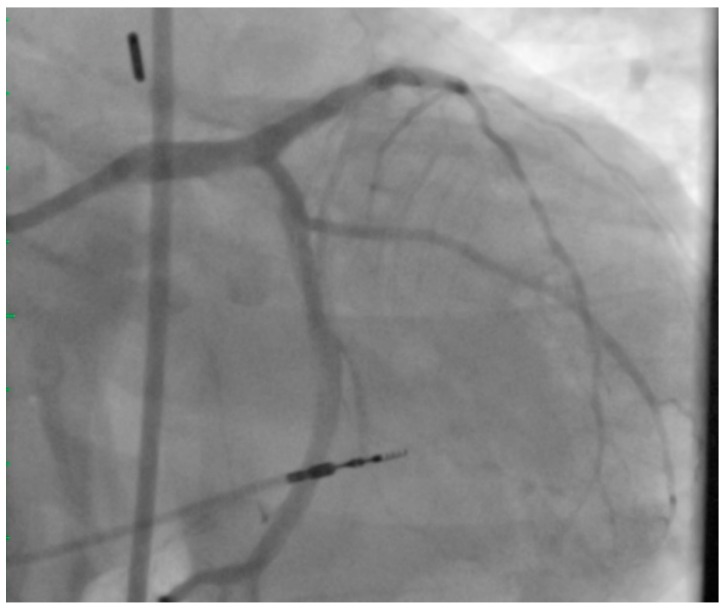
Final Coronary Angiogram. Excellent angiographic result after PCI of the LMCA with a 3.5 × 15 mm Resolute Onyx DES, post-dilated with a 4.0 mm noncompliant balloon. Additionally, PCI of the ostial CFX was completed using a modified T-stent technique with a 3.0 × 15 mm Resolute Onyx DES. Final kissing balloon inflations were performed.

The LMCA was stented with a 3.5 × 15 mm Medtronic Resolute Onyx DES (Medtronic, Minneapolis, MN, USA), with proximal optimization performed using a 4.0 × 8 mm non-compliant balloon. The CFX was subsequently re-crossed through the LMCA stent struts using the Asahi Sion wire. The CFX was predilated with a 2.5 mm compliant balloon. The ostial CFX was stented with a 3.0 × 15 mm Resolute Onyx DES using a modified T stent technique. Final kissing balloon inflations were performed in the LMCA and CFX with 4.0 mm and 3.0 mm noncompliant balloons respectively. A final 40 MHz IVUS was performed, revealing a minimal stent area of 13 mm^2^ (Figure 3) in the distal LMCA. The angiographic result (Figure 4) was excellent. The patient was seen in outpatient follow-up 4 months after UPLM PCI, no episodes of angina or dyspnea on exertion; major issue was mild cognitive impairment.

## 6. Conclusions

Durable clinical success in UPLM PCI is dependent on a number of procedural factors. Utilization of IVUS for procedure planning and evaluation of final stent area is established as the standard of care because of associated improved mortality, as well as reduced rates of ISR and stent thrombosis [30]. Two stent techniques, particularly DK Crush, are superior to single stent or provisional strategies. Given that the LMCA stenosis is often characterized by large plaque burden and calcification, a strategy of debulking for plaque modification can contribute to optimal stent expansion and achievement of stent areas that have been related to the best long term outcomes. In addition to endoluminal imaging with IVUS or optical coherence tomography, we believe that atherectomy should be applied more routinely in UPLM PCI.
